# Wisdom of the Wicked

**Published:** 1856-09

**Authors:** 


### WISDOM OF THE WICKED.

PASSING down Broadway the other day, we noticed two signs, and significant they were. They were signs that liquor drinking was not politic, and that the venders of it are on the look-out for means of sustaining themselves by devices creditable at once to their ingenuity and observation.

One of these signs was, "The Office"—the other "The Library." Does not the reader see the tact of the thing? It would be too vulgar to say, "Let's go and take a drink," or "walk over to the bar-room," which we suppose is a contraction of Barrel Room. But "Will you walk over to the 'Office?'" "Let's go to the LIBRARY."

Ah me! how much better it would be for humanity if the children of the light were as wise in their generation as the children of this world, and would study as hard all the little ways of luring men to virtue, which the wicked do in luring them to death.

